# Association Patterns in Saproxylic Insect Networks in Three Iberian Mediterranean Woodlands and Their Resistance to Microhabitat Loss

**DOI:** 10.1371/journal.pone.0122141

**Published:** 2015-03-26

**Authors:** Javier Quinto, María de los Ángeles Marcos-García, Cecilia Díaz-Castelazo, Víctor Rico-Gray, Eduardo Galante, Estefanía Micó

**Affiliations:** 1 Centro Iberoamericano de la Biodiversidad (CIBIO), Universidad de Alicante, Alicante, Spain; 2 Instituto de Ecología A.C. (INECOL), Xalapa, Veracruz, Mexico; Università degli Studi di Napoli Federico II, ITALY

## Abstract

The assessment of the relationship between species diversity, species interactions and environmental characteristics is indispensable for understanding network architecture and ecological distribution in complex networks. Saproxylic insect communities inhabiting tree hollow microhabitats within Mediterranean woodlands are highly dependent on woodland configuration and on microhabitat supply they harbor, so can be studied under the network analysis perspective. We assessed the differences in interacting patterns according to woodland site, and analysed the importance of functional species in modelling network architecture. We then evaluated their implications for saproxylic assemblages’ persistence, through simulations of three possible scenarios of loss of tree hollow microhabitat. Tree hollow-saproxylic insect networks per woodland site presented a significant nested pattern. Those woodlands with higher complexity of tree individuals and tree hollow microhabitats also housed higher species/interactions diversity and complexity of saproxylic networks, and exhibited a higher degree of nestedness, suggesting that a higher woodland complexity positively influences saproxylic diversity and interaction complexity, thus determining higher degree of nestedness. Moreover, the number of insects acting as key interconnectors (nodes falling into the core region, using core/periphery tests) was similar among woodland sites, but the species identity varied on each. Such differences in insect core composition among woodland sites suggest the functional role they depict at woodland scale. Tree hollows acting as core corresponded with large tree hollows near the ground and simultaneously housing various breeding microsites, whereas core insects were species mediating relevant ecological interactions within saproxylic communities, e.g. predation, competitive or facilitation interactions. Differences in network patterns and tree hollow characteristics among woodland sites clearly defined different sensitivity to microhabitat loss, and higher saproxylic diversity and woodland complexity showed positive relation with robustness. These results highlight that woodland complexity goes hand in hand with biotic and ecological complexity of saproxylic networks, and together exhibited positive effects on network robustness.

## Introduction

The incidence matrix of different species on different habitats exhibits specialized or non-random patterns of occurrences, such as nestedness [1–4] or modularity [5–6]. In addition, nestedness and modularity do not exclude each other, and spatially segregated modules may present a nested pattern [7–8]. Likewise, food webs behave similarly, and many types of trophic interactions display specialized patterns [9–11]. Their study may help to understand the ecological mechanisms underlying them and shed light on the relationship between interaction complexity and persistence [11–13]. For instance, trophic interactions with different outcomes for participant species or individuals across habitats in geographic space, determine network architecture resulting in different topologies and network persistence [10].

Biological diversity and complexity in food webs have great relevance for ecosystem stability, and a growing number of empirical studies demonstrate positive diversity-stability relationships [14–16]. The stability of ecosystems not only depends on the species richness, but also on trophic interactions that are capable of differential response, such as the degree of consumer specialization or differences in network complexity [15–19]. Therefore, trophic interactions are important mediators of the ecosystem complexity, and the removal of species can bring about complex and dramatic reorganizations of ecosystems, where consumers can modify, dampen or even reverse the directionality of diversity-productivity-stability linkages [20]. In spite of this, the mechanisms modelling network architecture and its relation with persistence are still scarcely understood in food webs describing species occurrences in geographic space, and the methodology available for classic networks of interaction between species may be helpful in this regard.

Species interactions and woodland characteristics are considered as pivotal factors modelling the composition of saproxylic [*sensu* 21–22] insect communities [23]. However, little is known about the spatial variation of saproxylic insect food webs in response to environmental characteristics. One of the most specialized saproxylic fauna within Mediterranean woodlands takes place within tree hollows [24–25]. In each tree hollow, different saproxylic species belonging to different feeding guilds are embedded within communities containing many species that may interact with one another (saproxylic predators, species competition or facilitation interactions) [10, 26] and with the physical environment of tree hollows and tree individuals (species exploiting woody resources and microsites, such as wood mould, water accumulations or saproxylic fungi) [27]. Differences at tree hollow microhabitat scale, such as hollow volume, hollow height to the ground or hollow opening areas, determine notorious differences in saproxylic guild composition among Mediterranean woodland sites [27], such that the study of how network patterns are established according to different woodlands is *per se* of great relevance, as well as for understanding their relation with the network persistence.

Here we qualitatively and quantitatively assessed for first time the occurrence and the variation of specialized network patterns in the tree hollow-saproxylic insect interaction among Mediterranean woodland sites. We selected three Mediterranean woodland sites in Cabañeros National Park (Spain), and the Coleoptera and Diptera (Syrphidae) sorted according to their feeding guild as study groups. Moreover, this is the first attempt to understand how saproxylic insect communities per woodland can be theoretically affected by the loss of their tree hollow microhabitats, analyzing their robustness from random and directed extinction simulations. We addressed the following questions: 1) Does the tree hollow-saproxylic interaction present specialized patterns at woodland scale? 2) Are there differences in the interacting patterns among woodland sites and which features are responsible of such differences? 3) How network properties are related with resistance to microhabitat loss?

## Material and Methods

### Study area and data collection

The permission was granted by the Spanish Ministry of Agriculture, Food and Environment and Cabañeros’ Administration. Field work was conducted in Cabañeros National Park (Spain), a protected natural area of 40,856 ha. The annual temperature average fluctuates between 12.9 and 15.6°C and annual precipitation averages between 500 and 750 mm. The park constituted a well-preserved Mediterranean landscape, where various woodland types appear inside a predominantly grassland and scrubland matrix [28–29]. We assessed one woodland site of three of the most representative Mediterranean woodland types in the Park: i) mixed deciduous oak woodland of *Quercus pyrenaica* Willd. and *Q*. *faginea* Lam. (39°21'20.37''N 4°23'42.15''W), where elevation ranged from 747 to 771 m, ii) riparian ash woodland of *Fraxinus angustifolia* Vahl. (39°26'50.34''N 4°33'49.32''W), with elevation ranging from 574 to 506 m, and iii) sclerophyllous woodland of *Q*. *rotundifolia* Lam. (39°26'45.48''N 4°31'51.90''W), where elevation ranged from 665 to 689 m [28]. In each woodland site we selected 30, 27, and 30 tree hollows, respectively. As sampling method we used emergence traps, a specific method to survey saproxylic communities inhabiting inside tree hollows (and its associated breeding microsites) [25, 30]. Samples were monthly collected from February 2009 to February 2010.

### Selected taxa, guild classification and characterization

We surveyed saproxylic Syrphidae (Diptera) and Coleoptera species. For species identification in many families we were assisted by international taxonomists (see Acknowledgments). We recorded 3926 individuals of Coleoptera belonging to 155 species and 39 families, and 461 individuals of Syrphidae belonging to 22 species (S1 Table). Each species was classified into saproxylic trophic guilds according to the ‘Frisbee’ data base [31]: xylophagous (X), saprophagous (including beetles and all species of hoverflies) (SA), saproxylophagous (SX), xylomycetophagous (XM) and predators (P). Saproxylic guilds were composed of 11 xylophagous species and 453 individuals, 56 saprophagous species and 1581 individuals, 30 saproxylophagous species and 1368 individuals, 29 xylomycetophagous species and 463 individuals, and 51 predator species and 522 individuals. Detailed information on species belonging to each saproxylic guild is shown in S1 Table.

### Network analysis and statistics

#### Network patterns

We used Aninhado [32] to analyze and compare the degree of nestedness. A nested pattern implies that more specialist species interact only with proper subsets of those species interacting with the more generalists [33]. We used the *NODF* estimator (nestedness based on overlap and decreasing fills) and performed CE null models [33] with 1000 replicates. CE considers that the probability of an interaction is proportional to the generalization level of both insect species and tree hollows, so allowing evaluating the influence of abundances to nestedness pattern [34]. This procedure allows the best estimation of the nestedness pattern because *NODF* is based on the nestedness of all pairs of columns and rows in the matrix [1]. In addition, we assessed the weighted nestedness (*WNODF*), a quantitative index for nestedness [35], and calculated the *WNODF* significance using RC null models [35]. This index is useful not only because it accurately estimates the nestedness of incidence matrices based on abundance data, but because it identifies the perfect nested pattern from the others. RC assigns individuals to matrix cells proportional to observed row and column abundance totals until, for each row and column, total abundances are reached [36].

In order to study the differences in modular pattern (the existence of clusters of species closely interacting with species of the same module) in each woodland site, we estimated the modularity using the modularity index *M* based on Barber’s algorithm (*Q*_*B*_) obtained through simulating annealing (*SA*), using Modular [37]. In order to test if our interaction networks had values of the modularity index *M* that were significantly higher than that of random networks, we performed 1000 randomizations to estimate their statistical significance. For a given partition of the nodes of a network into modules, the modularity *M* based on *Q*_*B*_ of this partition is [38–40]:

$$M=\sum_{m=1}^{N_{M}}\left[\frac{L_{m}}{L}-(\frac{K_{m}^{A}K_{m}^{B}}{L^{2}})\right]$$ (1)

where *N* *_M_* is the number of modules, *L* is the number of links in the complete network, *L*_*m*_ is the number of links between nodes in module *m*, and $K_{m}^{A}$ and $K_{m}^{B}$ are the sum of the degrees of all the A-nodes and all the B-nodes within module *m*. SA allows finding the optimal partition with largest modularity of the network into modules [41], being the most effective method to estimate the modularity in ecological networks [12, 42].

#### Interaction patterns

We used ‘bipartite’ package in R software [35] to draw bipartite graphs and to quantitatively assess the differences in network attributes among woodland sites. Network attributes considered were: links *L/S* (mean number of links per species, defined as the sum of links divided by the number of species), species degree (the sum of the diversity of links per species), interaction strength (sum of dependencies for each species), connectance *C* (the proportion of realised links of the total possible in each network, defined as the sum of links divided by the number of cells in the matrix), linkage density *LD* (a quantitative measure defined as the mean number of interactions per species), and *V-ratio* (variance ratio of species numbers to individual numbers within species for the higher trophic level: values larger than 1 indicate positive aggregation or association, values between 0 and 1 indicate disaggregation or competence of species).

#### Core/periphery

Using Ucinet 6 [43] we carried out categorical core/periphery analyses for bipartite graphs to study the number and position of species along the core-periphery gradient. Core/periphery structures are composed of a dense, cohesive core of species much better connected than others, and a sparse and unconnected periphery (hereafter named as core and peripheral species, respectively). In these structures, core nodes occur near the center and are proximate not only to each other but to all nodes in the network, and they used to be species with high species degree [44]. Ucinet 6 performs algorithms that allow locating each species position along the core-periphery gradient of the network, estimating a density value for each randomized matrix [43]. We performed 25 core-periphery randomizations for each woodland site, calculating the percentage that each species occurs within the core region. Because the large size of the target saproxylic networks, we considered two types of core species: a) core species falling into the core region more than 80% of the runs, and b) core species between 65–79% of the runs. Moreover, to easily locate core species (both tree hollows and insects) we indicated the position of each in the bipartite graphs obtained for each woodland site and ordered in decreasing number of interactions.

#### Simulations of microhabitat loss

Using the robustness function of ‘bipartite’ we simulated three different scenarios of tree hollow microhabitat loss in each woodland site as a measure of the tolerance of the saproxylic assemblages to the loss of their habitats [45]. This function calculates the area below the extinction curve generated by the removal of tree hollows, where R = 1 corresponds to a curve that decreases very mildly up until the point at which almost all tree hollows are eliminated, whereas with R = 0 the curve decreases abruptly as soon as any tree hollow is removed [46]. First, we performed a random deletion of tree hollows, secondly, according to its abundance, with least abundant tree hollows being erased first, and finally, following a sequence of elimination from the most to the least connected tree hollows.

## Results

We recorded 3926 individuals of Coleoptera belonging to 155 species and 39 families, and 461 individuals of Syrphidae belonging to 22 species (S1 Table). Saproxylic guilds were composed of 11 xylophagous species and 453 individuals, 56 saprophagous species and 1581 individuals, 30 saproxylophagous species and 1368 individuals, 29 xylomycetophagous species and 463 individuals, and 51 predator species and 522 individuals. Detailed information on species belonging to each saproxylic guild is presented in S1 Table.

The saproxylic assemblage at the deciduous oak woodland was composed of 137 species and 2343 individuals; the riparian ash woodland of 114 insect species and 1219 individuals; and the sclerophyllous oak woodland of 85 insect species and 825 individuals (see Table 1 for species composition of each guild per woodland site). The total abundance of each insect species per woodland site can be seen in S1 Table.

**Table 1:** Number of saproxylic insect species (S) and individuals (N) of each saproxylic guild per woodland site.

	DO	RA	SO
	S (N)	S (N)	S (N)
**X**	8 (233)	6 (111)	6 (109)
**SA**	45 (882)	31 (447)	29 (252)
**SX**	23 (644)	24 (412)	19 (312)
**XM**	24 (298)	18 (91)	13 (74)
**P**	37 (286)	35 (158)	18 (78)
**TOTAL**	137 (2343)	114 (1219)	85 (825)

DO: deciduous oak; RA: riparian ash; SO: sclerophyllous oak; X: xylophagous; SA: saprophagous; SX: saproxylophagous; XM: xylomycetophagous; P: predators.

### Differences in network patterns among woodland sites

All three woodland sites presented a tree hollow-insect interaction with significant qualitative and quantitative nested network pattern (p = 0.001) (Table 2). These were characterized by a low degree of nestedness, being the deciduous oak woodland which presented the highest nestedness value (*NODF* = 21.89, *WNODF* = 15), followed by the riparian ash woodland (*NODF* = 18.04, *WNODF* = 12.12), whereas the sclerophyllous oak showed the lowest nestedness value (*NODF* = 15.61, *WNODF* = 8.36). None of the studied tree hollow-saproxylic insect networks had a significant modular pattern (p > 0.05, most random matrices had lower *M* value than the real matrix).

**Table 2:** Network attributes modeling tree hollow-saproxylic interacting patterns per woodland site.

	DO	RA	SO
***N* (NODF)**	20.97	17.53	14.96
**WNODF**	15	12.12	8.36
***M* (SA)**	0.29	0.35	0.4
***L/S***	3.76	2.75	2.4
***C***	0.15	0.13	0.11
***LD***	10.58	8.64	7.3
***V-ratio***	6.56	4.83	4.46
**R RE**	0.71	0.67	0.66
**R DE1**	0.85	0.83	0.79
**R DE2**	0.52	0.46	0.48

DO: deciduous oak; RA: riparian ash; SO: sclerophyllous oak; *N* (NODF): nestedness using *NODF* estimator; *WNODF*: weighted nestedness; *M* (SA): modularity index using the simulating annealing procedure; *L/S*: links per species; *C*: connectance; *LD*: linkage density; *V-ratio*: variance ratio; R RE: robustness for a random extinction of tree hollows; R DE1: robustness for a directed extinction from the least to the most connected tree hollows; R DE2: robustness for a directed extinction from the most to the least connected tree hollows.

**Fig 1:**
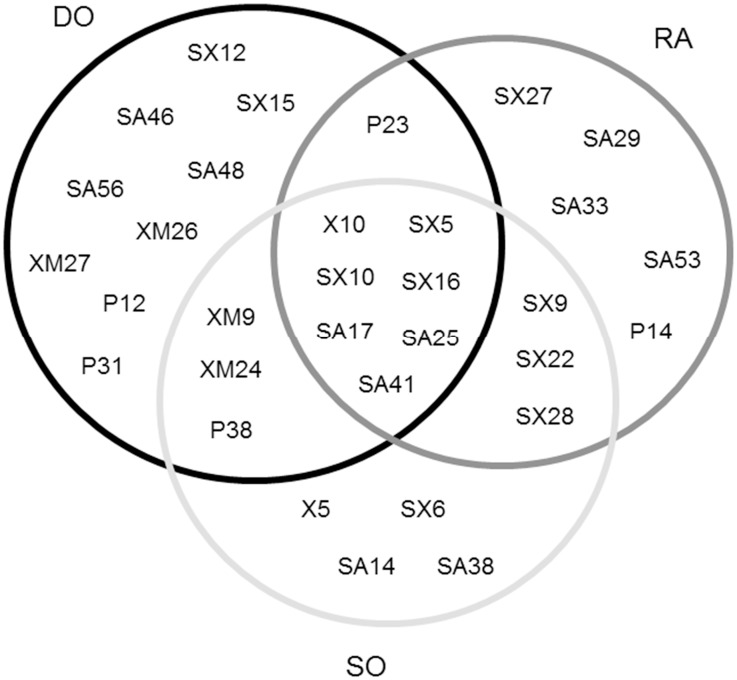
Core composition and position in bipartite graphs. Bipartite graphs showing the core composition and position in both tree hollow and insect trophic levels among woodland sites. Black square: tree hollow nodes in 65–79% of the randomizations. Grey square: tree hollow nodes in 80–100% of the randomizations. Black circle: insect nodes in 65–79% of the randomizations. Grey circle: tree hollow nodes in 80–100% of the randomizations.

The deciduous oak woodland exhibited the most densely interconnected network, presenting the highest values of links per species, connnectance and linkage density, followed by the riparian ash and the sclerophyllous oak woodlands (Table 2, Fig. 1). The most generalist insects were present in the three woodland sites, always reaching a high species degree and interaction strength, such as *Cryptophagus reflexus* (Cryptophagidae) (SX10) and *Xyleborus monographus* (Curculionidae Scolytinae) (X10), and presented a heterogeneous pattern of interconnections with both generalist and specialist tree hollows (high and low numbers of connections, respectively) (Fig. 1). Nevertheless, each woodland site presented particular insect species acting as important generalists or linking nodes, such as *Camptorhinus statua* (Curculionidae) (SX12) and *Soronia oblonga* (Nitidulidae) (SA48) in the deciduous oak woodland, *Ptinus timidus* (Ptinidae Ptininae) (SA53) in the riparian ash woodland, *Cetonia aurataeformis* (Cetoniidae) (SX5) for both deciduous oak and riparian ash woodland sites, and *Ischnomera xanthoderes* (Oedemeridae) (SX16) and *Alocerus moesiacus* (Cerambycidae) (SX4) in the sclerophyllous oak woodland. Regarding specialist species, the deciduous oak woodland exhibited the highest number, whereas the sclerophyllous oak woodland exhibited the lowest number of specialist species.

Moreover, *V-ratio* ecological indices exhibited low specialization in the global saproxylic-tree hollow matrix (Table 2), suggesting no common use of niche or disaggregation of species (such as competence or highly differentiated feeding habits), being the deciduous oak woodland the less specialized and more disaggregated in their pattern of interactions.

### Core nodes composition and distribution

**Fig 2:**
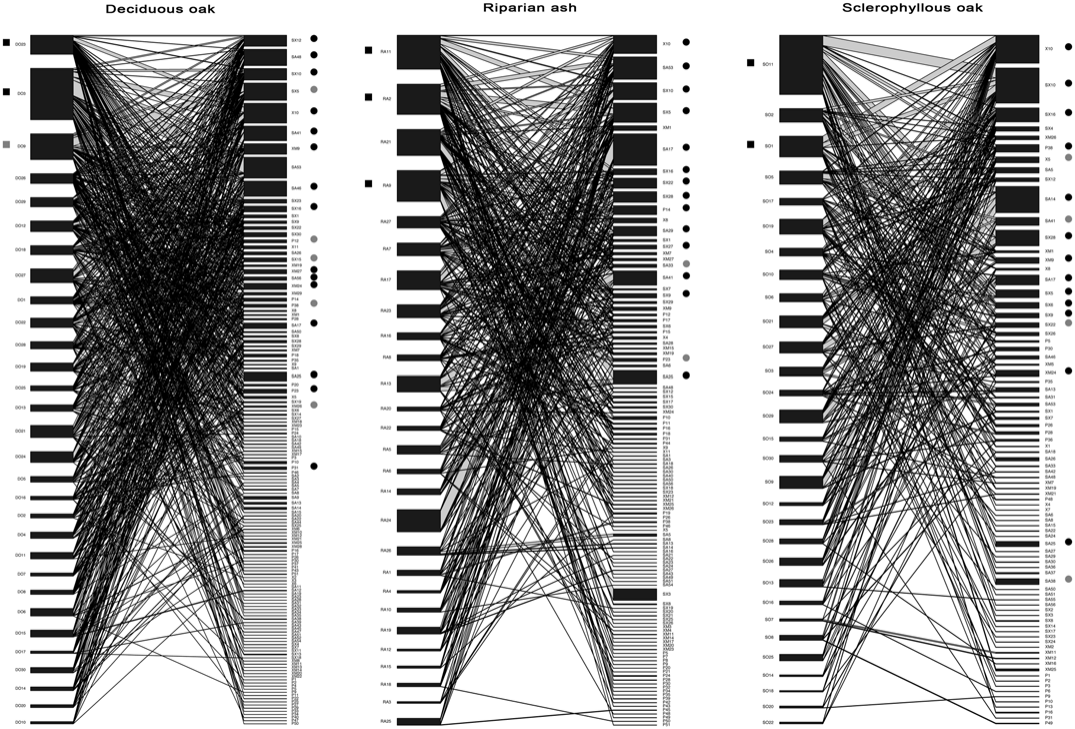
Differences in the insect core composition. Diagram depicting the insect core composition per woodland site (considering as core to those insect species falling into the core region more than 65% of the times), drawing both unique and shared insect core species among woodland sites. DO: deciduous oak; RA: riparian ash; SO: sclerophyllous oak. X: xylophagous; SA: saprophagous; SX: saproxylophagous; XM: xylomycetophagous; P: predators. Go to S1 Table to see which guild label corresponds with each insect species.

The number of tree hollows acting as core nodes were from two to three core tree hollows in each woodland site (Fig. 1), which corresponded with the most interconnected tree hollows. On the contrary, the number of insects acting as core nodes slightly varied according to woodland site (Fig. 2), being the deciduous oak woodland which had the highest number (20 core insects), followed by the riparian ash and the sclerophyllous oak woodland (17 core insects each). In addition, the composition of core insect species was quite different among woodlands sites, and only seven species were simultaneously core in the studied woodlands: *Cryptophagus reflexus* (SX10) and *C*. *micaceus* (SA25) (Cryptophagidae), (*Cetonia aurataeformis* (SX5) (Cetoniidae); *Ischnomera xanthoderes* (SX16) (Oedemeridae), *Myathropa florea* (SA17) (Syrphidae), *Xyleborus monographus* (X10) (Curculionidae), and *Prionocyphon serricornis* (SA41) (Helodidae). Moreover, the deciduous oak and the riparian ash woodlands also shared the predator *Gnathoncus communis* (P23) (Histeridae), whereas the sclerophyllous woodland shared three different core species with both the deciduous oak (*Cryptophagus scanicus* (Cryptophagidae) (XM9), *Mycetophagus quadriguttatus* (Mycetophagidae) (XM24), *Troglops furcatus* (Malachiidae) (P38)) and the riparian ash woodland (*Cryptophagus punctipennis* (SX9), *Scraptia testacea* (Scraptiidae) (SX22), *Mycetochara quadrimaculata* (SX28) (Tenebrionidae)). However, the deciduous oak showed the highest number of unique core species (nine species), followed by the riparian ash (five species), and the sclerophyllous oak woodland (four species). See Fig. 2 for further details in core-node composition per woodland site and the differences among woodlands.

For core assessment in bipartite graphs, the most interconnected nodes in tree hollow and saproxylic insect levels (higher species degree) not always corresponded with core nodes, and we observed that core category was more related with their heterogeneous pattern of interactions (Fig. 1). Moreover, many core insect nodes varied in their relative importance among woodland sites; that is, their position (number of different interactions) was markedly different according to the target woodland. On the other hand, core tree hollows not only had the highest species degree, but also they usually had the highest interaction strengths.

### Sensitivity to microhabitat loss

Saproxylic networks per woodland site showed high sensitivity to the loss of the most interconnected connected nodes (R: 0.46–0.52) (Table 2), which points to a rapid decline in the diversity of interactions after the removal of just a few of the most linked tree hollows. Conversely, they showed a relative tolerance to the elimination of the least interconnected tree hollows (R: 0.79–0.85), and most of the saproxylic insect species survived even when a high proportion of tree hollows were eliminated. On the other hand, the random extinction simulation of the tree hollows displayed an intermediate effect in the stability of the saproxylic communities (R: 0.66–0.71). For all extinction simulations, the saproxylic networks showed a higher robustness to the loss of tree hollows in the deciduous oak woodland, and as a noticeable data, the riparian ash was the most sensitive woodland to the directed simulation of loss of the most heterogeneously connected tree hollows.

## Discussion

Our results provide evidence that the woodland site substantially affects tree hollow-saproxylic insect networks, and shed light on how differences in microhabitat supply at woodland scale shape the architecture of interactions. Tree hollow-saproxylic insect networks per woodland site always presented a significant nested pattern. Woodlands displaying a higher degree of nestedness also were the most complex and densely connected, and the most aggregated in their pattern of occurrences/interactions. Such differences among woodland sites clearly defined different sensitivity to microhabitat loss, and those woodland sites which presented higher values better damped this impact throughout all the assessed extinction simulations. This study highlights the imperative need to identify and protect Mediterranean woodlands harboring high complexity of tree hollow microhabitats, and thus conserve the most diverse and ecologically robust saproxylic insect communities.

### Differences in network patterns among woodland sites

The saproxylic insect networks per woodland site exhibited a significantly nested pattern considering both qualitative (*NODF*) and quantitative data (*WNODF*), which means that higher diversity and density of interactions were established with the most generalist tree hollows/insects (the most densely and heterogeneously connected tree hollow/insects in each level).

All studied woodlands had a different size, species diversity (richness and abundance), disaggregation of species, and network complexity: level of connectance, links per species, linkage density and degree of qualitative and quantitative nestedness. The deciduous oak woodland presented higher values, in descending order followed by the riparian ash and the sclerophyllous oak woodland. The occurrence of a nested pattern in large trophic networks is commonly associated with higher heterogeneity in the link distribution [47]. Moreover, several types of woodland-dependent networks showing a nested structure have emphasized a high dependence on the habitat configuration; e.g. tree/fungus [4] or epiphyte-host networks [2]. Recent studies in the same Mediterranean woodlands have shown that woodland sites which hold higher microhabitat availability and heterogeneity (DO and RA) also determine higher saproxylic insect diversity, which is related with increased diversity and amount of trophic resources and breeding microsites for the different feeding guilds [27]. Thus a higher complexity of tree hollow microhabitats influences on the saproxylic diversity of tree hollows, which in turn promotes a higher complexity, heterogeneity and density of interactions, determining higher degree of nestedness in the deciduous oak woodland. Nonetheless, further research is necessary to unravel the variation of saproxylic food webs at different biogeographical scales, and within and among Mediterranean woodland types.

### The functional role of core species

The core nodes at tree hollow and saproxylic insect levels tended to have high species degree and interaction strengths, and showed higher complexity in their pattern of interconnections. For instance, tree hollows acting as core nodes were the ones more interconnected in each woodland site. They chiefly corresponded with those tree hollows displaying higher internal tree hollow volumes, simultaneously housing various breeding microsites, such as water accumulations, cetoniid feces content or the presence or activity of vertebrates, and whose heights to the ground were comprised between 0 and 70 cm. These characteristics have been recently recognized as modeling factors of saproxylic composition linked to tree hollows within Mediterranean woodlands [26–27], and as we show, they conspicuously influences on the structure of interactions by connecting the majority of the species of the network.

Saproxylic feeding guilds presented at least one species falling into the core region in the three woodland sites; however, the most representative guilds were the saproxylophagous and the saprophagous, together accumulating nearly 70% of the total of core insects (both considering core at 65–79% and higher than 80%). Quinto *et al*. [10] studied interacting patterns in saproxylic guilds inhabiting tree hollows and reported how guilds depending on woody substrates, such as the saprophagous/saproxylophagous guild, performed higher interaction diversity and complexity than insect-dependent guilds. Therefore, the higher proportion of the core within the saproxylophagous and the saprophagous guilds is strongly related with their dependence on woody resources within tree hollows. Furthermore, it is well documented how a few species may increase the whole community diversity because they hold higher interaction diversity and interaction strength [48–49], thereby determining an increase in the degree of nestedness [50–51]. Additionally, the increase of species diversity of certain functional groups can induce facilitative interactions, suggested as a key mechanism by which biodiversity enhances ecosystem functioning [51–53]. The latter emphasizes the main role that these particular trophic guilds have in the ecological dynamics at woodland scale and highlight their importance in determining the nested architecture of the saproxylic networks.

In addition, whereas the number of core insects was similar among woodland sites (17 to 20), the core identity varied on each, and only seven species were shared in all the three woodland sites. Two of those were *Cetonia aurataeformis* (Cetoniidae) and *Myathropa florea* (Syrphidae), which fell into the core region more than 80% of the times. Recently, it has been reported how the action of *C*. *aurataeformis* larvae in tree hollows produces a substrate that is chemically easier to decompose by other saproxylic insect species [54], which improves the development and fitness of many saprophagous syrphid species inhabiting tree hollows, such as *M*. *florea* [26]. Furthermore, *M*. *florea* is one of the most abundant syrphid species in the Mediterranean forest, mainly because their larvae feed on any type of fresh liquefied decaying plant matter, e.g. in water-filled tree hollows [55]. This suggests that *M*. *florea* is one of the first species which appear in tree hollows after the action of the common *C*. *aurataeformis*, and it could be acting as facilitator of other saproxylic species, but further empirical studies are needed. This is consistent with the findings of Fontaine *et al*. [56], who merged different types of networks and concluded that species traits that are important for one interaction are often directly or indirectly affected by another interaction. Biotic interactions may considerably modulate saproxylic communities via predation and interactive succession [23], parasitism of saproxylics [57], resource competition among species of the same feeding guild [10, 23] or, as mentioned above, through facilitation events [26, 58–59]. Hence, such core insect species composition in each woodland site may be evidencing the functional role they depict, mediating relevant ecological interactions within saproxylic communities, such as predation, competitive or facilitation interactions.

### Effect of woodland site on network robustness

Saproxylic networks showed a relative tolerance to both random extinctions and directed extinctions of the least interconnected tree hollows, leading a gradual decline in saproxylic species and interaction diversity. The fact that the bulk of the interactions were performed with the most ‘generalist’ tree hollows highlights their key role in maintaining the diversity and the ecological dynamic of saproxylic assemblages. However, saproxylic insects showing high dependence on the presence of scarce breeding microsites [24–25, 60] or presenting low dispersal ability [61] would be more sensitive to these kinds of microhabitat loss [62].

Conversely, saproxylic networks per woodland site showed high sensitivity to the loss of the most interconnected tree hollows, exhibiting a low extinction threshold at which the community collapses. This represents a critical scenario in which those tree hollows providing the most suitable microhabitats are removed, which would be comparable to an indiscriminate logging of larger trees in the woodland, representing the most severe but possible threat for the persistence of saproxylic assemblages. Moreover, the composition of core insect species mediating synergistic interactions [26, 58–59] would be drastically depleted, leading to an extinction succession difficult to predict (see [63]). Otherwise, ecosystem robustness can be considerably reduced by species extinctions of few of the most interconnected species [64–65], because this will quickly decrease saproxylic diversity and degree of nestedness. This would increase the degree of modularity by generating isolated subgroups of interacting tree hollows and insects (this could be ascertained by testing each network once again but e.g. reducing by half the tree hollows with higher degree).

A diversity/stability effect was observed among Mediterranean woodlands sites, and those woodlands housing the higher species and interaction diversity and complexity usually increased the robustness to the loss of tree hollow microhabitats, being the deciduous oak woodland the least sensitive to the elimination of the most connected tree hollows. However, the riparian ash woodland had fairly similar species/interaction diversity and was the most sensible to the loss of the most connected tree hollows (hollows used by the widest array of saproxylic insects), which reflects that a large number of unique species are taking place in a few tree hollows with higher degree. In general, higher species diversity, abundance of functional species, network complexity, degree of nestedness, or the distribution of interaction strengths, are positive signals of higher network stability and robustness in food webs [11, 13, 16, 45–46, 62–63, 66–68]. Nonetheless, diversity/stability relationships cannot be understood outside the context of the environmental drivers affecting both, and aspects as species-species interactions, food-web topology, and the tolerance to the habitat loss underlies diversity-stability relationships [17]. In summary, the woodland complexity goes hand in hand with the biotical and ecological complexity of saproxylic networks, and they jointly had positive effects on the network robustness.

## Supporting Information

S1 Table Species list, labels and abundances. Saproxylic species list, labels according to trophic guilds: saprophagous (SA); xylophagous (X), saproxylophagous (SX), xylomycetophagous (XM), predator (P) Species abundances in each woodland site: deciduous oak (DO), riparian ash (RA) and sclerophyllous oak (SO) woodland, and total abundance for each species.
(DOCX)
