# Supplementary material for: Association Patterns in Saproxylic Insect Networks in Three Iberian Mediterranean Woodlands and Their Resistance to Microhabitat Loss
Source: PLoS One. 2015 Mar 26;10(3):e0122141. doi: 10.1371/journal.pone.0122141 (PMC4374943; doi:10.1371/journal.pone.0122141)
Supplement: S1 Table — Saproxylic species list, labels according to trophic guilds: saprophagous (SA); xylophagous (X), saproxylophagous (SX), xylomycetophagous (XM), predator (P) Species abundances in each woodland site: deciduous oak (DO), riparian ash (RA) and sclerophyllous oak (SO) woodland, and total abundance for each species. (DOCX) [file pone.0122141.s001.docx]

**S1 Table. Species list, labels and abundances.** Saproxylic species list, labels according to trophic guilds: saprophagous (SA); xylophagous (X), saproxylophagous (SX), xylomycetophagous (XM), predator (P). Species abundances in each woodland site: deciduous oak (DO), riparian ash (RA) and sclerophyllous oak (SO) woodland, and total abundance for each species.

|  |  | **Label** | **DO** | **RA** | **SO** | **Total** |
| --- | --- | --- | --- | --- | --- | --- |
| DIPTERA |  |  |  |  |  |  |
| Syrphidae | *Brachyopa grunewaldensis* Kassebeer, 2000 | SA1 | 8 | 2 | 0 | 10 |
|  | *Brachypalpoides lentus* (Meigen, 1822) | SA2 | 2 | 0 | 0 | 2 |
|  | *Brachypalpus valgus* (Panzer, 1798) | SA3 | 2 | 2 | 0 | 4 |
|  | *Callicera aurata* (Rossi, 1790) | SA4 | 2 | 0 | 0 | 2 |
|  | *Callicera spinolae* Rondani, 1844 | SA5 | 3 | 10 | 16 | 29 |
|  | *Ceriana vespiformis* (Latreille, 1804) | SA6 | 0 | 8 | 1 | 9 |
|  | *Criorhina floccosa* (Meigen, 1822) | SA7 | 8 | 0 | 0 | 8 |
|  | *Criorhina pachymera* Egger, 1858 | SA8 | 2 | 1 | 1 | 4 |
|  | *Ferdinandea aurea* Rondani, 1844 | SA9 | 24 | 0 | 0 | 24 |
|  | *Ferdinandea cuprea* (Scopoli, 1763) | SA10 | 3 | 0 | 0 | 3 |
|  | *Ferdinandea fumipennis* Kassebeer, 1999 | SA11 | 1 | 0 | 0 | 1 |
|  | *Ferdinandea ruficornis* (Fabricius, 1775) | SA12 | 6 | 0 | 0 | 6 |
|  | *Mallota cimbiciformis* (Fallen, 1817) | SA13 | 17 | 3 | 13 | 33 |
|  | *Mallota dusmeti* Andreu, 1926 | SA14 | 18 | 2 | 77 | 97 |
|  | *Mallota fuciformis* (Fabricius, 1794) | SA15 | 2 | 0 | 1 | 3 |
|  | *Milesia crabroniformis* (Fabricius, 1775) | SA16 | 0 | 1 | 0 | 1 |
|  | *Myathropa florea* (Linnaeus, 1758) | SA17 | 46 | 128 | 29 | 203 |
|  | *Myolepta difformis* Strobl in Czerny & Strobl, 1909 | SA18 | 4 | 3 | 3 | 10 |
|  | *Myolepta dubia* (Fabricius, 1805) | SA19 | 1 | 0 | 0 | 1 |
|  | *Myolepta obscura* Becher, 1882 | SA20 | 4 | 0 | 0 | 4 |
|  | *Sphiximorpha subsessilis* (Illiger in Rossi, 1807) | SA21 | 0 | 1 | 0 | 1 |
|  | *Spilomyia digitata* (Rondani, 1865) | SA22 | 3 | 1 | 2 | 6 |
| COLEOPTERA |  |  |  |  |  |  |
| Aderidae | *Aderus populneus* (Panzer, 1796) | SX1 | 25 | 9 | 4 | 38 |
|  | *Cnopus minor* (Baudi, 1877) | SX2 | 0 | 0 | 1 | 1 |
|  | *Otolelus neglectus* (Jacquelin du Duval, 1863) | SX3 | 0 | 47 | 1 | 48 |
| Biphyllidae | *Diplocoelus fagi* Guérin-Ménéville, 1838 | XM1 | 8 | 19 | 6 | 33 |
| Buprestidae | *Acmaeodera degener 14-punctata* (Scopoli, 1763) | X1 | 0 | 0 | 2 | 2 |
|  | *Eurythyrea quercus* (Herbst, 1780) | X2 | 1 | 0 | 0 | 1 |
| Carabidae | *Lamprias cyanocephalus* (Linne, 1758) | P1 | 1 | 0 | 1 | 2 |
|  | *Syntomus foveatus* (Geoffroy in Fourcroy, 1785) | P2 | 1 | 0 | 1 | 2 |
|  | *Trechus obtusus* Erichson, 1837 | P3 | 4 | 0 | 1 | 5 |
| Cerambycidae | *Alocerus moesiacus* (Frivaldsky, 1838) | SX4 | 2 | 0 | 13 | 15 |
|  | *Cerambyx welensii* (Küster, 1846) | X3 | 3 | 0 | 0 | 3 |
|  | *Prinobius myardi* Mulsant, 1851 | X4 | 0 | 11 | 1 | 12 |
|  | *Stictoleptura trisignata* (Fairmaire, 1852) | X5 | 20 | 1 | 17 | 38 |
|  | *Trichoferus fasciculatus* (Faldermann, 1837) | X6 | 1 | 0 | 0 | 1 |
| Cerylonidae | *Cerylon histeroides* Fabricius, 1792 | P4 | 1 | 0 | 0 | 1 |
| Cetoniidae | *Cetonia aurataeformis* Curti, 1913 | SX5 | 144 | 78 | 23 | 245 |
|  | *Potosia cuprea* (Fabricius, 1775) | SX6 | 6 | 11 | 17 | 34 |
|  | *Potosia opaca* (Fabricius, 1787) | SX7 | 1 | 6 | 7 | 14 |
| Ciidae | *Cis striatulus* Mellie, 1845 | XM2 | 0 | 0 | 1 | 1 |
|  | *Cis villosulus* Marsham, 1802 | XM3 | 0 | 1 | 0 | 1 |
| Clambidae | *Calyptomerus* sp | XM4 | 0 | 1 | 0 | 1 |
|  | *Clambus* sp | XM5 | 0 | 0 | 4 | 4 |
| Cleridae | *Opilo domesticus* (Sturm, 1837) | P5 | 1 | 1 | 5 | 7 |
| Cryptophagidae | *Atomaria pusilla* (Paykull, 1798) | SA23 | 1 | 1 | 0 | 2 |
|  | *Cryptophagus aurelii* Otero, 2009 | SX8 | 14 | 4 | 3 | 21 |
|  | *Cryptophagus cylindrus* Kiesenwetter, 1858 | XM6 | 4 | 0 | 0 | 4 |
|  | *Cryptophagus dentatus* (Herbst, 1793) | XM7 | 11 | 10 | 2 | 23 |
|  | *Cryptophagus distinguendus* Sturm, 1845 | SA24 | 0 | 1 | 1 | 2 |
|  | *Cryptophagus fallax* Balfour-Browne 1953 | XM8 | 1 | 0 | 0 | 1 |
|  | *Cryptophagus micaceus* Rey, 1889 | SA25 | 73 | 54 | 15 | 142 |
|  | *Cryptophagus punctipennis* Brisout de Barneville, 1863 | SX9 | 22 | 18 | 14 | 54 |
|  | *Cryptophagus reflexus* Rey, 1889 | SX10 | 96 | 69 | 103 | 268 |
|  | *Cryptophagus saginatus* Sturm, 1845 | SA26 | 24 | 2 | 10 | 36 |
|  | *Cryptophagus scanicus* (Linnaeus, 1758) | XM9 | 88 | 14 | 17 | 119 |
| Curculionidae | *Camptorhinus simplex* Seidlitz, 1866 | SX11 | 2 | 0 | 0 | 2 |
|  | *Camptorhinus statua* (Rossi, 1790) | SX12 | 92 | 3 | 11 | 106 |
|  | *Gasterocercus hispanicus* Alonso-Zarazaga, Jover & Micó, 2009 | XM10 | 2 | 0 | 0 | 2 |
| Platypodinae | *Platypus cylindrus* (Fabricius, 1792) | X7 | 0 | 0 | 1 | 1 |
| Scolytinae | *Xyleborinus saxesenii* (Ratzeburg, 1837) | X8 | 11 | 20 | 6 | 37 |
|  | *Xyleborus dryographus* (Ratzeburg, 1837) | X9 | 6 | 3 | 0 | 9 |
|  | *Xyleborus monographus* (Fabricius, 1792) | X10 | 165 | 74 | 82 | 321 |
| Dasytidae | *Aplocnemus brevis* (Rosenhauer, 1856) | P6 | 0 | 0 | 1 | 1 |
|  | *Aplocnemus consobrinus* (Rosenhauer, 1856) | P7 | 0 | 1 | 0 | 1 |
|  | *Aplocnemus limbipennis* Kiesenwetter, 1865 | P8 | 0 | 1 | 0 | 1 |
|  | *Mauroania bourgeoisi* (Pic, 1894) | P9 | 1 | 1 | 1 | 3 |
| Dermestidae | *Anthrenus angustefasciatus* Ganglbauer, 1904 | SA27 | 0 | 2 | 1 | 3 |
|  | *Anthrenus festivus* Erichson, 1846 | SA28 | 1 | 7 | 0 | 8 |
|  | *Anthrenus minutus* Erichson, 1846 | SA29 | 0 | 39 | 1 | 40 |
|  | *Anthrenus verbasci* (Linnaeus, 1767) | SA30 | 0 | 3 | 2 | 5 |
|  | *Attagenus incognitus* Hava, 2003 | SA31 | 1 | 0 | 4 | 5 |
|  | *Attagenus schaefferi* (Herbst, 1792) | SA32 | 1 | 0 | 0 | 1 |
|  | *Attagenus trifasciatus (Fabricius, 1787)* | SA33 | 1 | 13 | 3 | 17 |
|  | *Dermestes bicolor* Fabricius, 1781 | SA34 | 1 | 0 | 0 | 1 |
|  | *Dermestes erichsonii* Ganglbauer, 1904 | SA35 | 1 | 0 | 0 | 1 |
|  | *Dermestes frischii* Kugelann, 1792 | SA36 | 0 | 0 | 1 | 1 |
|  | *Dermestes hispanicus* Kalik, 1952 | SA37 | 0 | 0 | 4 | 4 |
|  | *Dermestes undulatus* Brahm, 1790 | SA38 | 5 | 0 | 17 | 22 |
|  | *Orphilus niger* (Rossi, 1790) | SA39 | 2 | 0 | 0 | 2 |
| Dynastidae | *Oryctes nasicornis* (Linnaeus, 1758) | SX13 | 7 | 0 | 0 | 7 |
| Elateridae | *Ampedus aurilegulus* (Schaufuss, 1862) | P10 | 21 | 13 | 1 | 35 |
|  | *Ectamenogonus montandoni* Buysson, 1888 | P11 | 2 | 5 | 0 | 7 |
|  | *Elater ferrugineus* Linnaeus, 1758 | P12 | 25 | 8 | 0 | 33 |
|  | *Elathous platiai* Zapata & Sánchez-Ruiz, 2007 | P13 | 0 | 0 | 1 | 1 |
|  | *Ischnodes sanguinicollis* (Panzer, 1793) | P14 | 27 | 36 | 0 | 63 |
|  | *Lacon punctatus* (Herbst, 1779) | P15 | 4 | 7 | 0 | 11 |
|  | *Limoniscus violaceus* (Müller, 1821) | SA40 | 0 | 2 | 0 | 2 |
|  | *Megapenthes lugens* (Redtenbacher, 1842) | P16 | 9 | 8 | 3 | 20 |
|  | *Melanotus dichrous* (Erichson, 1841) | SX14 | 11 | 0 | 1 | 12 |
|  | *Procraerus tibilais* (Boisduval & Lacordaire, 1835) | P17 | 2 | 10 | 0 | 12 |
| Endomychidae | *Mycetaea hirta* (Marsham, 1802) | XM11 | 2 | 2 | 3 | 7 |
|  | *Symbiotes gibberosus* (Lucas, 1849) | XM12 | 2 | 2 | 2 | 6 |
| Eucinetidae | *Nycteus meridionalis* Laporte de Castelnau, 1835 | XM13 | 1 | 0 | 0 | 1 |
| Helodidae | *Prionocyphon serricornis* (Müller, 1821) | SA41 | 122 | 58 | 23 | 203 |
| Histeridae | *Abraeus perpusillus* (Marsham, 1802) | P18 | 24 | 8 | 0 | 32 |
|  | *Aeletes atomarius* (Aubé, 1842) | P19 | 0 | 6 | 0 | 6 |
|  | *Atholus corvinus* (Germar, 1817) | P20 | 16 | 1 | 0 | 17 |
|  | *Atholus duodecimstriatus* (Schrank, 1781) | P21 | 0 | 1 | 0 | 1 |
|  | *Eubrachium hispidulum* (Bremi-Wolf, 1855) | P22 | 2 | 0 | 0 | 2 |
|  | *Gnathoncus communis* (Marseul, 1862) | P23 | 34 | 13 | 0 | 47 |
|  | *Gnathoncus nannetensis* (Marseul, 1862) | P24 | 10 | 4 | 0 | 14 |
|  | *Hetaerius ferrugineus* (Olivier, 1789) | P25 | 1 | 0 | 0 | 1 |
|  | *Kissister minimus* (Laporte, 1840) | P26 | 2 | 2 | 7 | 11 |
|  | *Margarinotus brunneus* (Fabricius, 1775) | P27 | 1 | 0 | 0 | 1 |
|  | *Margarinotus merdarius* (Hoffmann, 1803) | P28 | 12 | 2 | 8 | 22 |
|  | *Margarinotus uncostriatus* (Marseul, 1854) | P29 | 2 | 0 | 0 | 2 |
|  | *Merohister ariasi* (Marseul, 1864) | P30 | 2 | 1 | 11 | 14 |
|  | *Paromalus flavicornis* (Herbst, 1792) | P31 | 23 | 4 | 1 | 28 |
|  | *Platylomalus complanatus* (Panzer, 1797) | P32 | 0 | 1 | 0 | 1 |
|  | *Platylomalus gardineri* (Scott, 1913) | P33 | 1 | 0 | 0 | 1 |
|  | *Platysoma filiforme* (Erichson, 1834) | P34 | 1 | 1 | 0 | 2 |
| Laemophloeidae | *Cryptolestes ferrugineus* (Stephens, 1831) | SA42 | 5 | 0 | 2 | 7 |
|  | *Laemophloeus nigricollis* Lucas, 1849 | XM14 | 1 | 1 | 0 | 2 |
|  | *Placonotus testaceus* (Fabricius, 1787) | SA43 | 1 | 1 | 0 | 2 |
| Latridiidae | *Corticaria inconspicua* Wollaston, 1860 | XM15 | 5 | 5 | 0 | 10 |
|  | *Corticaria obscura* Brisout, 1863 | SA44 | 2 | 0 | 0 | 2 |
|  | *Dienerella ruficollis* (Marsham, 1802) | XM16 | 0 | 0 | 1 | 1 |
|  | *Enicmus brevicornis* (Mannerheim, 1844) | XM17 | 3 | 1 | 0 | 4 |
|  | *Enicmus rugosus* (Herbst, 1793) | XM18 | 4 | 0 | 0 | 4 |
|  | *Latridius assimilis* Mannerheim, 1844 | XM19 | 14 | 9 | 2 | 25 |
| Leiodidae | *Agathidium nigriceps* Brisout de Barneville, 1872 | XM20 | 1 | 1 | 0 | 2 |
| Lucanidae | *Dorcus parallelepipedus* (Linnaeus, 1758) | SX15 | 34 | 9 | 0 | 43 |
| Malachiidae | *Anthocomus fenestratus* Linder, 1864 | P35 | 11 | 1 | 4 | 16 |
|  | *Axinotarsus marginalis* (Laporte, 1840) | P36 | 0 | 0 | 6 | 6 |
|  | *Hypebaeus albifrons* (Fabricius, 1775) | P37 | 3 | 0 | 0 | 3 |
|  | *Troglops furcatus* Abeille, 1885 | P38 | 25 | 5 | 22 | 52 |
| Melandryidae | *Orchesia micans* (Panzer, 1794) | XM21 | 4 | 2 | 2 | 8 |
| Melyridae | *Falsomelyris granulata* (Fabricius, 1792) | P39 | 0 | 2 | 0 | 2 |
| Mycetophagidae | *Litargus balteatus* LeConte, 1856 | XM22 | 1 | 0 | 0 | 1 |
|  | *Litargus connexus* (Fourcroy, 1785) | XM23 | 9 | 2 | 0 | 11 |
|  | *Mycetophagus quadriguttatus* Müller, 1821 | XM24 | 51 | 5 | 17 | 73 |
| Nitidulidae | *Carpophilus* sp | SA45 | 1 | 0 | 0 | 1 |
|  | *Amphotis marginata* (Fabricius, 1781) | P41 | 2 | 0 | 0 | 2 |
|  | *Epuraea fuscicollis* (Stephens, 1835) | SA46 | 127 | 0 | 10 | 137 |
|  | *Epuraea ocularis* Fairmaire, 1849 | SA47 | 2 | 0 | 0 | 2 |
|  | *Soronia oblonga* Brisout de Barneville, 1863 | SA48 | 136 | 3 | 2 | 141 |
| Oedemeridae | *Ischnomera xanthoderes* (Mulsant, 1858) | SX16 | 48 | 24 | 41 | 113 |
| Ptinidae |  |  |  |  |  |  |
| Anobiinae | *Oligomerus brunneus* (Olivier, 1790) | X11 | 26 | 2 | 0 | 28 |
| Dorcatominae | *Dorcatoma vaulogeri agenjoi* Español, 1978 | XM25 | 8 | 3 | 6 | 17 |
|  | *Rhamna semen* Peyerimhoff de Fontenelle, 1913 | XM26 | 13 | 3 | 11 | 27 |
|  | *Stagetus elongatus* (Mulsant & Rey, 1861) | SX17 | 0 | 6 | 1 | 7 |
|  | *Stagetus micoae* Viñolas, 2011 | SX18 | 1 | 2 | 0 | 3 |
| Ptininae | *Dignomus irroratus* (Kiesenwetter, 1851) | SA49 | 3 | 1 | 0 | 4 |
|  | *Ptinus bidens* Olivier, 1790 | SA50 | 10 | 2 | 1 | 13 |
|  | *Ptinus hirticornis* Kiesenwetter, 1867 | SA51 | 1 | 1 | 1 | 3 |
|  | *Ptinus spitzyi* Villa & Villa, 1838 | SA52 | 1 | 0 | 0 | 1 |
|  | *Ptinus timidus* Brisout de Barneville, 1866 | SA53 | 174 | 91 | 9 | 274 |
| Ptiliidae | sp 1 | XM27 | 36 | 10 | 0 | 46 |
| Rhizophagidae | *Rhizophagus unicolor* (Lucas, 1846) | P40 | 1 | 0 | 0 | 1 |
| Scraptiidae | *Anaspis regimbarti* Schilsky, 1890 | SX19 | 10 | 1 | 0 | 11 |
|  | *Pentaria defarguesi* Abeille de Perrin, 1885 | SX20 | 0 | 1 | 0 | 1 |
|  | *Scraptia ophthalmica* Mulsant, 1856 | SX21 | 0 | 2 | 0 | 2 |
|  | *Scraptia testacea* Allen, 1940 | SX22 | 25 | 41 | 14 | 80 |
| Scydmaenidae | Goniaceritae tribe sp 2 | P42 | 0 | 1 | 0 | 1 |
|  | *Scydmaenus cornutus* Motschulsky, 1845 | P43 | 2 | 1 | 0 | 3 |
|  | *Stenichus godarti* (Latreille, 1806) | P44 | 0 | 5 | 0 | 5 |
| Silvanidae | *Ahasverus advena* (Waltl, 1834) | SA54 | 1 | 1 | 0 | 2 |
|  | *Oryzaephilus surinamensis* (Linnaeus, 1758) | SA55 | 0 | 0 | 1 | 1 |
|  | *Silvanus bidentatus* (Fabricius, 1792) | P45 | 0 | 2 | 0 | 2 |
|  | *Uleiota planata* (Linnaeus, 1761) | P46 | 5 | 2 | 0 | 7 |
| Tenebrionidae | *Corticeus fasciatus* Fabricius, 1790 | P47 | 1 | 0 | 0 | 1 |
|  | *Eledonoprius armatus* (Panzer, 1799) | XM28 | 7 | 0 | 0 | 7 |
|  | *Probaticus anthracinus* (Allard, 1876) | SX23 | 32 | 4 | 1 | 37 |
|  | *Probaticus granulatus* (Allard, 1876) | SX24 | 0 | 0 | 1 | 1 |
|  | *Stenohelops sublinearis* (Kraatz, 1870) | SX25 | 3 | 1 | 0 | 4 |
|  | *Tenebrio punctipennis* Seidlitz, 1896 | SA56 | 29 | 3 | 1 | 33 |
| Alleculinae | *Isomira hispanica* Kiesenwetter, 1870 | SX26 | 0 | 1 | 10 | 11 |
|  | *Mycetochara linearis* (Latreille, 1804) | SX27 | 4 | 16 | 0 | 20 |
|  | *Mycetochara quadrimaculata* (Latreille, 1804) | SX28 | 21 | 43 | 46 | 110 |
|  | *Prionychus fairmairei* (Reiche, 1860) | SX29 | 8 | 9 | 0 | 17 |
|  | *Pseudocistela ceramboides* (Linnaeus, 1761) | SX30 | 36 | 7 | 0 | 43 |
| Trogossitidae | *Temnochila caerulea* (Olivier, 1790) | P48 | 0 | 1 | 2 | 3 |
|  | *Tenebroides marrocanus* Reitter, 1884 | P49 | 0 | 1 | 2 | 3 |
| Zopheridae | *Colobicus hirtus* (Rossi, 1790) | P50 | 1 | 1 | 0 | 2 |
|  | *Colydium elongatum* (Fabricius, 1787) | P51 | 5 | 1 | 0 | 6 |
|  | *Endophloeus marcovichianus* (Piller & Mitterpacher, 1783) | XM29 | 22 | 0 | 0 | 22 |
